# Dry Heating of Cowpea Flour below Biopolymer Melting Temperatures Improves the Physical Properties of Bread Made from Climate-Resilient Crops

**DOI:** 10.3390/foods11111554

**Published:** 2022-05-25

**Authors:** Stefano Renzetti, Ine Heetesonne, Ruth T. Ngadze, Anita R. Linnemann

**Affiliations:** 1 Wageningen Food and Biobased Research, Wageningen University & Research, Bornse Weilanden 9, 6708 WG Wageningen, The Netherlands; 2 Food Quality and Design Group, Wageningen University & Research, Bornse Weilanden 9, 6708 WG Wageningen, The Netherlands; ine.heetesonne@wur.nl (I.H.); ruth.ngadze@wur.nl (R.T.N.); anita.linnemann@wur.nl (A.R.L.)

**Keywords:** cowpea flour, dry heating, climate-resilient crops, pasting properties, thermal properties, bread

## Abstract

Improving the technological functionality of climate-resilient crops (CRCs) to promote their use in staple foods, such as bread, is relevant to addressing food and nutrition security in Africa. Dry heating of cowpea flour (CPF) was studied as a simple technology to modulate CPF physicochemical properties in relation to bread applications. For this purpose, the melting behavior of cowpea starch and proteins in CPF was first studied and modeled using Flory–Huggins theory for polymer melting. Next, dry-heating conditions were investigated based on the predicted biopolymer melting transitions in CPF to be well below starch and protein melting. The pasting properties (i.e., peak viscosity, final viscosity, breakdown and setback) of CPF could be selectively modulated depending on temperature-time combinations without altering the thermal behavior (i.e., melting enthalpies) of CPF. Water-binding capacity and soluble solids decreased with the increased severity of the temperature-time combinations. Dry-heated CPF added to CRC-based bread significantly improved crumb texture. In particular, dry heating at 100 °C for 2 h provided bread with the highest crumb softness, cohesiveness and resilience. The positive effects on the crumb texture could be largely related to enhanced starch integrity, as indicated by a reduction in breakdown viscosity after treatment. Overall, dry heating of CPF under defined conditions is a promising technology for promoting the use of CPF as a techno-functional and protein-rich ingredient in bread-type products.

## 1. Introduction

Currently, the rapidly increasing population in Sub-Saharan Africa (SSA) largely relies on the import of wheat to provide convenient and affordable food such as refined wheat bread [1]. With wheat imports reaching almost 60% of the internal demand [2], this situation poses a major economic and food security problem.

Alternative grains and legumes that are indigenous in SSA have traditionally been used by local communities to prepare meals. Several of these crops, such as sorghum, millets, cassava and cowpeas, are also climate-resilient crops (CRCs). Fostering a predominant use of locally available CRCs in the production of attractive, affordable and nutritious bread provides a sustainable solution for the urgent food security issue by promoting agri-food development, reducing imports and providing income for smallholder farmers [1,3]. In view of climate changes, the rediscovery and exploitation of CRCs are crucial to improving food security, as they have enhanced tolerance to biotic and abiotic stress [4,5].

Cowpea is a starchy legume grown on the African continent, which is seen as a major cash crop by Central and West African farmers, with an estimated 200 million people consuming the legume daily. It requires very few inputs, making it a valuable crop for resource-poor farmers [6]. In addition, cowpea can grow in soils of up to 85% sand, in low rainfall and shade [7]. This makes it compatible as an intercrop with sorghum [8]. Cowpeas can be processed into flour, which is high in carbohydrates and protein. Cowpea proteins are rich in glutamic acid, aspartic acid, and lysine, making cowpeas interesting to the human diet [6]. Additionally, cowpea flour is a source of bioactive compounds, such as polyphenols and peptides, dietary fiber and resistant starch, and vitamins and minerals [9,10]. Therefore, using cowpea in composite flours with sorghum and cassava to improve the technological and nutritional properties of bread-type products is relevant to addressing food nutrition and security issues in SSA.

Dry heating is an interesting technique for improving the functionality of flours by physical modification. It is safe, simple and requires little cost, time and no use of chemicals [11]. In recent years, research has been performed on the effect of dry heating on starches (often in combination with gums), but the modification of flour using dry-heating treatments has rarely been studied [12]. Nonetheless, these studies have shown that dry heating has a more significant effect on the structural properties of flour than individual constituents such as starches. This has been attributed to the presence of non-starch components, such as protein and lipids [13]. Studies with yam and millet flour showed significant changes in flour functionality, reporting an increase in pasting viscosities after treatment [12,13]. Positive effects of toasting or dry heating of pulses have also been reported in pea flour (i.e., increased gel-forming abilities) [14], chickpeas (i.e., higher specific bread volume) [15] and yellow peas (i.e., improved flavor and inactivation of antinutritional compounds) [16]. However, to the best of our knowledge, no studies have been performed on the dry heating of cowpea flour.

Against this background, the aim of this study was to evaluate the effects of dry-heating conditions on the functionality of cowpea flour (CPF) in relation to bread applications. To implement a systematic approach, the melting behavior of proteins and starch in the CPF was first studied as a function of moisture content. Cowpea proteins were isolated and studied to discriminate the complex melting transitions in the CPF. Based on the insights into protein denaturation and starch gelatinization temperatures, defined temperature and moisture conditions were selected for the dry-heating treatments. Next, thermal analysis, water binding capacity and pasting properties of the native and dry-heated CPF were studied to assess the impact on CPF functionality. Finally, the ability of the dry-heated CPF to improve the physical properties of breads made from CRCs (i.e., sorghum, cassava and cowpea) composite flours was assessed by performing baking trials.

## 2. Materials and Methods

### 2.1. Materials

Red, non-tannin, King Korn superfine sorghum (locally known as *mabele*) meal was obtained from a local supermarket in Pretoria. Red (variety Glenda) cowpea seeds were sourced from Agrinawar Agricol Pty (Pretoria, South Africa) and milled as whole grains at ProCorn Mills (Edenvale, South Africa). Sorghum contained 10.2% protein, 11.5% dietary fiber (of which 2.3% was soluble) and 73.1% starch. CPF contained 23.9% protein (of which 18.4% was soluble at native pH), 24.1% dietary fiber (of which 7.7% was soluble) and 39.7% starch.

Cassava starch (93.2% starch, 0.3% protein and 2.1% soluble fiber) was from DADTCO (Dutch Agricultural Development and Trading Company, Inhambane, Mozambique). Psyllium husk powder (88% dietary fiber, 3% proteins) was from Unilecithin (Sharjah, United Arab Emirates). Dry yeast (Mauripan red, AB Mauri, Dordrecht, The Netherlands), salt and sucrose (both locally sourced) were used for the breadmaking trials.

### 2.2. Methods

#### 2.2.1. Cowpea Protein Isolation

CPF was first defatted by suspending 300 g flour in 700 mL petroleum ether and stirring for 2 h at room temperature. Then, the flour was left to sediment and the clear yellow supernatant was removed. This procedure was repeated four times. The defatted suspension was filtered over folded paper and left at room temperature for 48 h to dry. Protein isolation was performed by isoelectric point precipitation following [17]. Briefly, defatted cowpea flour was dispersed in distilled water at a 1:5 ratio. The pH of the suspension was adjusted to pH 9 with 1N NaOH. The mixture was stirred at room temperature for 20 min while adjusting the pH to 9. The suspension was then centrifuged at 4000× *g* for 20 min at 4 °C. The pellet was suspended once again at pH 9 and stirred for 20 min and centrifuged. The supernatants were then combined, and the pH was adjusted to pH 4 with 1 M HCL. The sample was centrifuged as before and washed 3 times with distilled water to remove salts and then neutralized to pH 7. The samples were freeze-dried and then ground into powder with a mortar to provide cowpea protein concentrate (CPPC). The protein content of CPPC was measured in duplicate by combustion (Dumas method, AOAC method 990.03) using a Flash EA 1112 protein analyzer (Thermo Fisher Scientific, Waltham, MA, USA). The detected nitrogen content was converted to protein content using 6.25 as the conversion factor.

#### 2.2.2. SDS-PAGE of CPF and CPPC

CPF and CPPC (10 mg/mL) were dissolved in 0.06 M tris buffer at pH 6.8 containing 2% SDS, 20% glycerol, 10% beta-mercaptoethanol and bromophenol blue, mixed, stirred and heated for 10 min at 95 °C. After centrifugation at 4000× *g* for 20 min at 4 °C, the supernatant was applied to the gel (anykDa precast gel, Bio-Rad, Hercules, CA, USA). The gel was stained with Coomassie blue.

#### 2.2.3. Melting Transitions of Native Cowpea Flour and Cowpea Protein Isolate at Different Levels of Hydration

Thermal analysis was performed with a TA Instruments type Q200 Differential Scanning Calorimeter (DSC) to measure starch gelatinization and protein denaturation. For CPPC and CPF, protein denaturation and starch gelatinization temperatures were studied at sample concentrations of 10 to 95% in distilled water, following a procedure recently reported [18]. About 6 mg of the sample was weighed in stainless steel cups and water was added. Cups were then hermetically sealed and left to hydrate overnight. Samples were then analyzed in a DSC by first equilibrating at −5 °C for 5 min and then heating up at a rate of 5 °C/min to 160 °C, 180 °C or 230 °C depending on hydration level [18]. The onset temperature of starch gelatinization and protein denaturation (T_onset_), peak temperature (T_peak_), end temperature (T_end_) and gelatinization/denaturation enthalpy were determined using the analysis tool available in the Universal Analysis software. Experiments were performed in triplicate.

From the melting data, the state diagram of CPPC first and then the one from CPF was obtained by applying the Flory–Huggins (FH) theory for biopolymer melting, as recently applied to starch and proteins in water and sugar solutions [18,19,20,21]:

(1) $$\frac{1}{T_{m}}-\frac{1}{T_{m}^{{}^{\circ}}}=\frac{R}{\Delta H_{U}}\frac{v_{U}}{v_{W}}\left[\Phi_{water}-\chi_{p}\cdot \Phi_{water}{}^{2}\right]$$

where *Φ_water_* is the volume fraction of water, $T_{m}$ (K) is the melting temperature of the biopolymer in the system under consideration, $T_{m}^{{}^{\circ}}$ (K) the melting temperature of the dry biopolymer, $\Delta H_{U}$ (kJ/mol) is the melting enthalpy per mole of the repeat unit of the biopolymer, $v_{U}$ is the molar volume of the biopolymer repeat unit, $v_{w}$ is the molar volume of water, *χ_p_* is the FH solvent-biopolymer interaction parameter and *R* is the universal gas constant. It should be noted that in FH theory, the crystalline phase of biopolymers does not absorb water. The volume fraction of the biopolymer in the rubbery state is computed based on its degree of crystallinity ξ [19].

#### 2.2.4. Moisture Sorption Behavior of Cowpea Protein Isolate and Cowpea Flour

The moisture sorption behavior of the CPPC and CPF samples was determined in duplicate according to [22] using an automatic multi-sample moisture sorption analyzer SPSx-11l (Projekt Messtechnik, Ulm, Germany).

The sorption properties were modeled using the Flory–Huggins Free Volume (FHFV) theory [23,24]:

(2) $$lna_{w}=\ln\left(\Phi_{w}\right)+\left(1-1/N_{s}\right)\left(1-\Phi_{w}\right)+\chi_{s}{\left(1-\Phi_{w}\right)}^{2}+F\left(\Phi_{w}\right)$$

where *N_s_* is the ratio of molar volumes of solute versus water (ν*_s_*/ν*_w_*), χ_s_ is the FH interaction parameter of the solute with water. $F\left(\Phi_{w}\right)$ accounts for structural relaxation in the glassy state and the changes in hydrogen bonding between water and polymer in the semi-dilute regime. $F\left(\Phi_{w}\right)$ is calculated from:

(3) $$F\left(\Phi_{w}\right)=\{\begin{array}{c} \;0,\;if\;T\geq T_{g}\\ -M_{w}y_{s}^{2}\frac{\Delta C_{p,w}}{RT}\frac{dT_{g}}{dy_{s}}\frac{T-T_{g}}{T_{g}},\;\;if\;T\leq T_{g} \end{array}$$

where *M_w_* is the molecular weight of water, *y_s_* is the mass fraction of the solute and *T* is the actual temperature. $T_{g}$ is the glass transition temperature of the water–biopolymer mixture, as computed from the Couchman-Karasz equation:

(4) $$T_{g}=\frac{y_{w}T_{g,w}\Delta C_{p,w}+\sum_{i}y_{s,i}T_{g,s,i}\Delta C_{p,s,i}}{y_{w}\Delta C_{p,w}+\sum_{i}y_{s,i}\Delta C_{p,s,i}}$$

Δ*C_p,i_* is the change in heat capacity at the glass transition, and *T_g,i_* is the glass transition temperature in the dry state. *y_i_* represents the weight fraction of the polymer or water. We have used Δ*C_p,s_* = 0.425 kJK^−1^ following [24].

$\frac{dT_{g}}{dy_{s}}$ is computed from [19]:

(5) $$\frac{dT_{g}}{dy_{s}}=\frac{\Delta C_{p,s}C_{p,w}\left(T_{g,w}-T_{g,s}\right)}{{\left(y_{w}\Delta C_{p,w}+y_{s}\Delta C_{p,s}\right)}^{2}}$$

#### 2.2.5. Dry-Heating Treatments of Cowpea Flour

The CPF was first equilibrated at 20 °C in a humidity-controlled cabinet (Weiss Technik Nederland, Tiel, The Netherlands) set at 50% RH, as this related to a moisture content of around 10%. This was done by sifting a thin layer of flour into open aluminum pans (the maximum height of the flour was about 1 cm) and equilibrating for 24 h. The flour was mixed once during equilibration. The water activity and moisture content of the flour were checked to confirm equilibration. After equilibrating, the flour was packed in heat-resistant bags in portions of 50 g and vacuum-sealed (Henkovac International, ‘s-Hertogenbosch, The Netherlands) to keep the moisture conditions constant during heating.

The CPF was heated for 2, 4, 24 and 48 h at 80 °C, 100 °C and 120 °C in a stove (Memmert GmbH, Schwabach, Germany). After heating, a rolling pin was used to break up the clumps of the flour. The codes given to the different variations are a combination of the temperature and time (e.g., 80C-2h).

#### 2.2.6. Thermal Analysis of Dry-Heated Cowpea Flours

For the dry-heated CPF samples, thermal analysis was performed at flour concentrations (on a dry matter basis) of 20% in distilled water following the same DSC scanning protocol previously described.

#### 2.2.7. Water Binding Capacity and Soluble Solids of Native and Dry-Heated CPF

The water-binding capacity (WBC) of the native and dry-heated CPF samples was determined in triplicate, according to a modified version of the protocol of [25]. Flours (0.4 g on dry basis) were placed in 5 mL Eppendorf tubes and 3.6 g of distilled water was added during vigorous stirring. After mixing on a vortex, the samples were left to shake at room temperature for 20 min on a Multi Reax Vortex (Heidolph Instruments GmbH, Schwabach, Germany). Then, the samples were centrifuged for 10 min at 5000× *g* using an Avanti J-26XP High Speed Centrifuge (Beckman Coulter, Indianapolis, IN, USA). The supernatant was collected, and the pellet was drained for 15 min at an angle of 45° and then weighed. WBC was expressed as follows:

$$\mathrm{WBC}\;\left(g/g\right)=\frac{\left(\mathrm{wet}\;\mathrm{pellet}\;\left(g\right)-\mathrm{dried}\;\mathrm{pellet}\;\left(g\right)\right)}{\left(\mathrm{dried}\;\mathrm{pellet}\;\left(g\right)\right)\;}$$

For the determination of the soluble solids, the collected supernatant was dried overnight in an oven at 105 °C. The soluble solids were expressed as follows:

$$\mathrm{Soluble}\;\mathrm{solids}\;\left(g/g\right)=\frac{\left(\mathrm{dry}\;\mathrm{weight}\;\mathrm{of}\;\mathrm{supernatants}\;\left(g\right)\right)}{\left(\mathrm{dry}\;\mathrm{weight}\;\mathrm{of}\;\mathrm{flour}\;\left(g\right)\right)}$$

#### 2.2.8. Rapid Visco Analyzer (RVA) of Native and Treated Flours

The pasting behavior of all the CPF samples was investigated using a Rapid Visco Analyser Super 4 (Perten, Hägersten, Sweden). Briefly, sample suspensions of 8% dry matter (dm) in water were prepared for a total weight of 25.0 g. The samples were subjected to a time–temperature profile. The initial stirring speed was 960 rpm at 50 °C for 60 s. Then, the stirring speed was decreased to 160 rpm, while the temperature was increased to 95 °C within 3 min 42 s. Samples were then held at 95 °C for 2 min 30 s minutes and cooled to 50 °C within 3 min 48 s. Finally, samples were held at 50 °C for 2 min. Experiments were performed in duplicate. Data analysis was performed using TCW3 software (Perten, Hägersten, Sweden). The parameters obtained from RVA were peak viscosity (PV), hold viscosity (HV), final viscosity (FV), breakdown viscosity (BD), set back (SB) and pasting temperature (T_paste_).

#### 2.2.9. Bread-Making Procedure

A reference bread dough formulation consisting of the CRC flours sorghum, cassava and CPF was developed based on preliminary trials. The preliminary trials consisted of modifying a commercial gluten-free bread formulation based on oat flour as provided by Bakkerij Wiltink (Doetinchem, The Netherlands) to include the CRC ingredients. The final recipe consisted of 50 g sorghum flour, 50 g cassava flour, 9.5 g CPF, 8 g psyllium husk powder, 5.5 g dry yeast, 4 g rapeseed oil, 4 g sucrose, 2.5 g salt and 119 g water.

In total, about 200 g of dry ingredients were added to a Brabender Farinograph (Brabender GmbH & Co. KG, Duisburg, Germany) and pre-mixed at a speed of 63 rpm. The mixing chamber was pre-set at 20 °C. Then water was slowly added during mixing, which was performed for 6 min. After mixing, the dough was divided and shaped manually, and put into three greased baking tins (158 mL volume; 10 cm × 4.5 cm × 3.5 cm). Each tin contained 105 g of dough. These tins were put in fermentation cabinets at 30 °C and 85% RH. The proofing time was defined as the time needed by 50 g of dough to reach a CO_2_ production of 90 mL. The CO_2_ production was determined using a Risograph (National Manufacturing, Lincoln, NE, USA). After proofing, the doughs were put in a swing oven at 180 °C for 40 min. During the first minute, steam was injected twice to regulate moisture content. After baking, the breads were cooled at room temperature for 40 min, sealed in plastic low-density polyethylene bags and stored at room temperature until further analysis one day after baking. Baking tests were performed in triplicate.

#### 2.2.10. Bread Quality Evaluation

Loaf volume was determined on 2 loaves from each baking test, with a rapeseed displacement according to the AACC method 10–05.01. In total, 6 measurements were performed per variation. Specific volume (SV) was calculated as loaf volume divided by loaf weight (mL/g).

Crumb texture was measured by means of Texture Profile Analysis using a TA-XT2i Texture Analyzer from Stable Micro Systems (Godalming, Surrey, UK) with a 30 kg load cell and a 75 mm compression plate and performed as described by [25]. In total, 12 measurements were performed per bread type.

The moisture content of the breadcrumbs (5 g sample) was measured according to the AACC standard method 44-15.02 by drying overnight in aluminum dishes in an oven at 105 °C. The filled dishes were cooled for 1 h in a desiccator before weight was determined. In total, 6 measurements were performed per bread type.

#### 2.2.11. Statistical Analysis

Statistical evaluation (analysis of variance, ANOVA, with Tukey’s-Test as post-hoc test at a significance level of *p* < 0.05) was performed with SPSS (IBM, version 25, Chicago, IL, USA), and correlation analysis and regression were performed with Rstudio (RStudio version 1.1.463, Inc., Boston, MA, USA).

## 3. Results and Discussion

### 3.1. Protein Profile of CPF and CPPC

**Figure 1:**
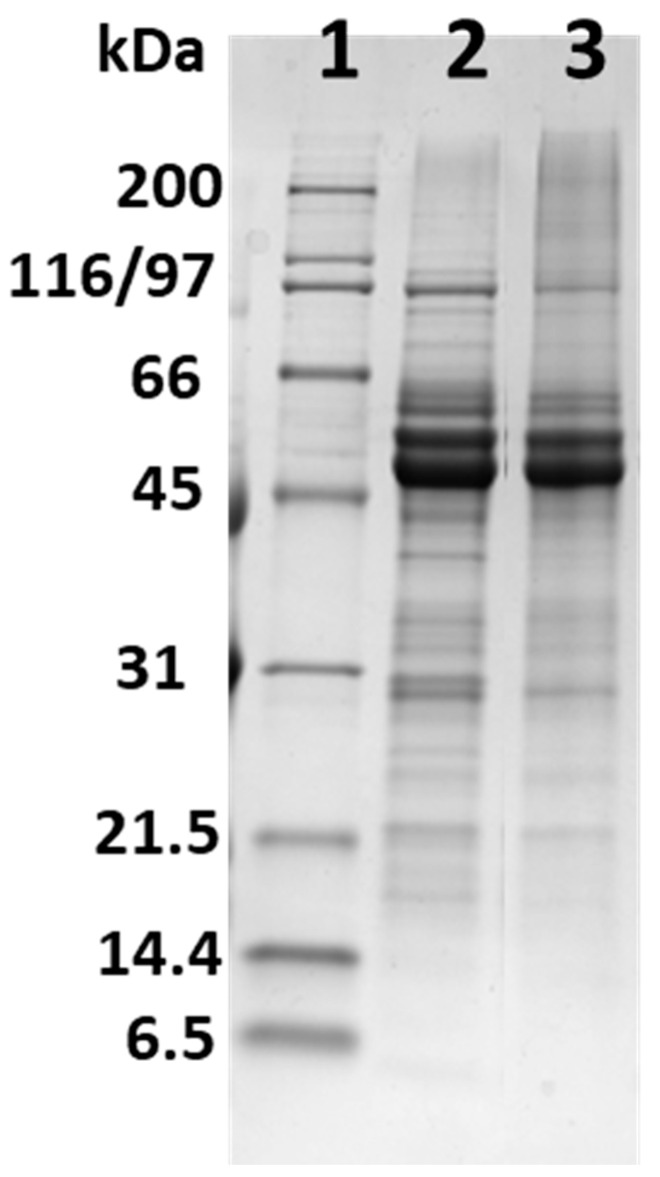
SDS-PAGE of CPF (2) and CPPC (3) with molecular weight markers (1). Proteins were stained with Coomassie blue.

CPF and CPPC were examined by SDS-PAGE (Figure 1). The protein profile of CPF showed main bands at about 50, 52 and 60 kDa and minor bands around 30 kDa and below, as previously reported [26]. The CPPC contained 81.5% proteins, with main bands at 50–60 kDa. These were in the typical molecular mass range of 7S storage proteins, in agreement with data previously reported [26,27].

### 3.2. Characterization of the Thermal and Sorption Properties of CPF and CPPC

**Figure 2:**
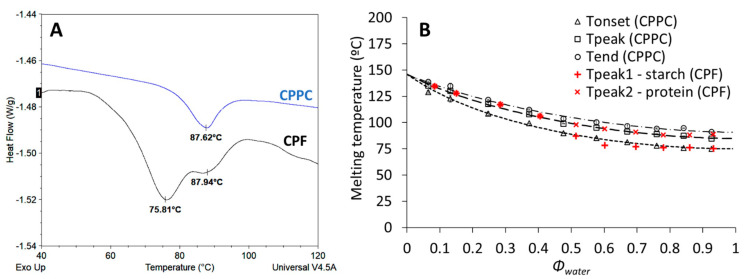
DSC thermograms of CPPC and CPF showing the melting transitions of proteins (i.e., 88 °C) and starch (i.e., 76 °C) (**A**); melting temperatures of proteins in CPPC (black symbols) and of proteins and starch in CPF (red symbols) as a function of *Φ*_*water*_. The dotted black lines represent the FH model describing the melting transitions (**B**).

The thermal properties of CPF and CPPC were studied to describe protein denaturation and starch gelatinization temperatures as a function of moisture content. Based on this information, dry-heating temperature conditions could be better defined in relation to the biopolymer phase transitions. In excess water (flour and protein concentration of 20%), CPF showed two endothermic peaks at 76 and 88 °C, while CPPC showed one peak at 88 °C (Figure 2A). The denaturation temperature of CPPC was in agreement with those reported for 7S storage proteins from cowpeas and soy [26]. For CPF, the peak at 76 °C was in agreement with the gelatinization temperature of isolated cowpea starch [28].

From the comparative analysis of CPF and CPPC, peaks related to starch and protein denaturation could be assigned. The denaturation behavior, i.e., T_onset_, T_peak_ and T_end_, of CPPC derived from the analysis of DSC thermograms was plotted as a function of *Φ_water_* (Figure 2B). An increase in denaturation temperature could be observed with decreasing moisture content. T_onset_, T_peak_ and T_end_ data were modeled using FH theory as recently applied to osther proteins, including soy [18,20,29]. The fitted parameters were T_m,0_, *ΔH_U_*, and the degree of crystallinity ξ [19]. Via regression, we obtained T_m,0_ = 419.1 (K), ΔH_U,Tonset_ = 49.6 kJ/mol, ΔH_U,Tpeak_ = 59.2 kJ/mol, ΔH_U,Tend_ = 66.7 kJ/mol, ξ_onset_ = 0.22, ξ_peak_ = 0.08 and ξ_end_ = 0.

The denaturation temperature of cowpea proteins in CPF as a function of *Φ_water_* was similar to CPPC, thus confirming the interpretation of the DSC thermograms for the flour. For *Φ_water_* ≥ 0.5, the peak related to starch gelatinization in CPF followed a similar trend as the T_onset_ of CPPC denaturation. By comparing the DSC thermograms of CPF and CPPC (Figure 2A), it could be observed that the T_onset_ of CPPC denaturation and the T_peak_ of starch overlapped. For *Φ_water_* < 0.5, the T_peak_ of starch gelatinization and that of protein denaturation merged into a single peak in CPF (data not shown).

**Figure 3:**
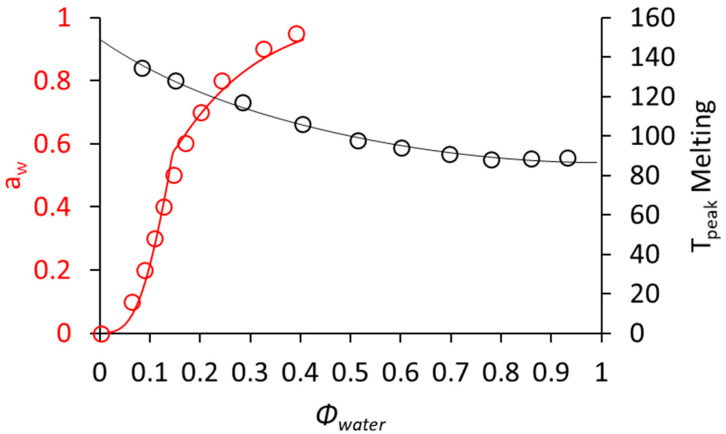
Peak temperature of CPPC denaturation (black circles) and moisture sorption behavior of CPPC (red circles) as a function of *Φ_water_* as described by the FH model (Equation (1)) and the FHFV model (Equation (2)), respectively. From the FHFV model, the T_peak_ of CPPC after equilibration at a specific *a_w_* can be predicted using the FH model.

The sorption properties of CPF and CPPC were similar in the water activity range from 0 to 0.9, while a slightly higher moisture absorption was detected at an *a_w_* of 0.95 for CPF compared to CPPC (Supplementary Figure S1). Given their similarities, only the isotherm for CPPC was plotted as a function of *Φ_water_* together with the T_peak_ of protein denaturation (Figure 3). The sorption data were fitted using the FHFV theory as recently applied to soy protein isolate [24]. Considering that CPPC is composed of 7S storage proteins, the dry *T_g_* was assumed to be 386K, similar to what was reported for 7S soy globulins [30]. Sorption isotherms were fitted with respect to χ_1_ obtaining a value of 0.7. This is lower than the 0.91 value reported for the soy protein isolate [24]. It should be noted that the lower χ_1_, the more hygroscopic the behavior of the material. At an *a_w_* of 0.9, CPPC was able to absorb 0.3 g water/g dry solids, which is substantially higher than the 0.24 g water/g dry solids reported for the soy protein isolate [24].

The material properties described in Figure 3 were used to select the temperature conditions for dry heating in relation to the biopolymer phase transitions in CPF. As previously noted, at *Φ_water_* < 0.5 protein and starch transitions occur around the same temperatures. For an *a_w_* of 0.5, the T_peak_ of melting was predicted to be about 132 °C. In recent studies, dry heating of cereal and legume flours has been shown to be more effective in enhancing the pasting and gelling properties compared to similar treatments on the isolated starches [12,13,31]. However, these studies were conducted at high temperatures (130 °C) for short times (2–4 h), without defined relations to the melting transitions of the biopolymers in the materials. Furthermore, moisture conditions were not defined even though the amount of water affects melting temperatures. Therefore, to test the functionalization of cowpea flour in well-defined conditions, dry heating was performed at the selected temperatures of 80, 100 and 120 °C, corresponding to 60%, 75% and 90% of the melting temperature of cowpea proteins and starch when pre-equilibrated at 50% relative humidity. The treatments covered both short (2 and 4 h) and long (24 and 48 h) times.

### 3.3. Characterization of Thermal Properties of Dry-Heated CPF

The melting transitions in the dry-heated CPF were studied in comparison to the native flour. All treated samples showed an endothermic transition in temperature ranges similar to the native flour. Remarkably, the melting enthalpy for dry-heated CPF was generally higher than for the native one (Supplementary Table S1). However, only samples 100C-48h and 120C-48h showed a significant increase compared to the native CPF (*p* < 0.05). With regards to the melting temperatures, the main effect of the treatments seemed to be a smoothing of the peak related to protein denaturation (i.e., T_peak,2_) (Supplementary Figure S2), which resulted in the disappearance of T_peak,2_ for the most severe temperature-time combinations, i.e., 100C-48h, 120C-24h and 120C-48h (Supplementary Table S1).

The limited changes in the melting enthalpies may be explained by annealing of the starch and partial unfolding of proteins, since the temperature conditions were well below the peak temperature of starch gelatinization and protein denaturation. Phillips and co-workers [32] also reported preservation of starch birefringence when cowpeas were treated in the temperature range of 70–130 °C, although their treatment time was less than two hours.

### 3.4. WBC and Solubility of Native and Dry-Heated CPF

In general, dry heating resulted in a significant reduction in both solubility and WBC compared to the native CPF (Table 1). At 80 °C, a significant difference in solubility and WBC was observed between short (2 and 4 h) and prolonged (24 and 48 h) treatment times, with the latter showing the largest reduction compared to the native CPF. The treatments at 120 °C showed the lowest values in solubility and WBC, regardless of the treatment time. All samples treated at 100 °C showed a significant reduction in solubility and WBC compared to the native CPF, but no clear trends were observed with regard to treatment time.

The amount of soluble solids in CPF (i.e., 29.1%) was well in agreement with the compositional analysis, with soluble proteins and soluble fiber accounting for 26.1% of soluble solids. Hence, soluble proteins accounted for about 70% of soluble solids in the native CPF, the rest being mainly soluble fiber. The dry-heating treatments resulted in a reduction of soluble solids as high as 43.6% at 120 °C. The progressive reduction in the melting peak related to protein denaturation with increasing severity of the dry-heating conditions suggests that the soluble protein was largely affected by the dry heating. In fact, a progressive reduction in soluble proteins was earlier reported in cowpea meal with increasing dry-heating temperatures from 70 to 130 °C [32]. It would be expected that the insolubilization of soluble solids such as proteins would result in an increased ability to bind water. Phillips and co-workers [32] also reported a decrease in WBC of cowpea flour, which they attributed to the increased hydrophobicity of proteins or annealing of starch. Our DSC results indicating the denaturation of protein with limited changes in melting enthalpies suggest that both mechanisms could contribute. On the other hand, it should be noted that in CPF, the starch is tightly covered with protein material [33,34], which limits the water-binding and swelling power of the flour [34]. In a protein matrix, water binding is controlled by swelling, which is inversely related to cross-linking density [24]. From this standpoint, the partial denaturation and aggregation of the cowpea proteins can reduce WBC due to the inverse relationship between protein cross-linking and water-holding ability. Additionally, these aggregates can act as a barrier on the starch surface, thus further limiting hydration.

### 3.5. Pasting Properties of Native and Dry-Heated CPF

**Figure 4:**
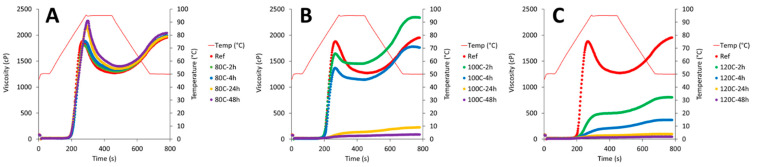
RVA pasting profiles of dry-heated CPF for temperature conditions of 80 °C (**A**), 100 °C (**B**) and 120 °C (**C**) following 2, 4, 24 and 48 h of treatment time. Profiles are compared to the native flour reference (Ref in red).

The dry-heating treatments largely modulated the pasting properties of CPF depending on the specific conditions (Figure 4). Significant effects were observed for all pasting parameters (*p* < 0.05; Table 2). Prolonged dry heating at low temperatures (i.e., 80C-24h and 80C-48h) significantly increased PV, while for the higher temperatures, a progressive reduction in PV with increasing treatment time was observed. In particular, the treatments at 100 °C and 120 °C for 24 and 48 h resulted in almost no viscosity build-up during the temperature cycle. The HV was significantly enhanced for 80C-24h, 80C-48h and 100C-2h. Interestingly, for 100C-2h that was associated with a significant decrease in BD compared to native CPF, while a significant increase in BD was obtained for samples 80C-24h and 80C-48h. A significant increase in FV was obtained only for sample 100C-2h, while all other treatments at 100 °C and 120 °C showed a significant reduction in FV. The SB significantly increased only for 100C-2h, while all other samples showed a significant reduction compared to native CPF, except for 80C-2h and 80C-4h.

The pasting behavior of flour and starch is largely dictated by granule rigidity and granule swelling [35]. PV is reached when the rate of swelling is equal to the rate of breakdown during heating [36]. As the rate of granule rupture exceeds that of swelling, a decrease in paste viscosity (i.e., BD) is observed and the starch molecules disperse in the aqueous phase [37]. Changes in protein structures around the starch granules resulting from the dry heating of CPF can significantly affect pasting behavior [38,39,40]. Protein aggregation around the starch granules can limit starch swelling, thus reducing paste viscosity. At the same time, the aggregated proteins make the swollen granules less susceptible to breakdown, either by conferring strength to the swollen granules or by reducing the degree of swelling. These mechanisms are consistent with the observed changes in the pasting profiles of the dry-heated CPF samples. The protein denaturation observed in the DSC traces of dry-heated CPF with the concomitant reduction in soluble material suggests the strengthening of the protein barrier tightly covering the starch granules. However, it should be noted that these mechanisms cannot fully describe the changes observed in the samples treated at 80 °C for 24 and 48 h, where an increase in PV and BD were observed. An increase in paste viscosity and BD has been observed for dry heating of flours such as rice and proso millet [12,13]. These were associated with both protein–protein and protein–starch interactions, as protein dissociation with a reducing agent had only limited effect in reducing the paste viscosity of the dry-heated flours. The degree of starch crystallinity was also enhanced by heat treatment. In our study, the pasting behavior of samples 80C-24h and 80C-48h may be the result of increased granule rigidity conferred by protein aggregation and enhanced starch crystallinity. In the concentrated regime conditions of the RVA, starch granules cannot swell to their maximum because of space restrictions [41]. Under such conditions, the rheology of the system is dictated by the rigidity of the suspended particles [42]. With further protein aggregation caused by the more intensive heat treatments, swelling likely became the limiting factor, thus causing the observed reductions in paste viscosity. Overall, it is interesting to note that choices of temperature-time combinations as related to melting transitions allowed the selective modulation of specific pasting parameters in this study, as compared to a general increase in the pasting profile of dry-heated flours [12,13].

### 3.6. Selection of Dry-Heated CPF for Testing in a Tin-Bread Application

**Figure 5:**
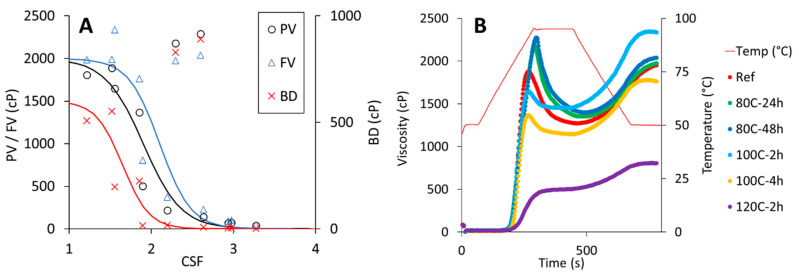
Pasting properties (i.e., PV = peak viscosity, FV = final viscosity and BD = breakdown) as a function of the combined severity factor CSF (**A**). Pasting profiles of native (Ref) and dry-heated CPF that were selected for the breadmaking experiments (**B**). For the dry-heated CPF samples, the temperature (C) and time (h) treatment conditions are indicated by the codes.

To gain better insights into the effect of dry-heating conditions on CPF functionality, pasting properties and WBC were analyzed as a function of the combined severity factor (CSF). CSF can be conceptualized as a variable that represents the combination of time and temperature, being related to the extent of the reaction from a starting kinetic model. For hydrothermal treatments, it has been generally proposed [43]:

$$CSF=e^{\frac{E_{a}}{R}\frac{\left(T-T_{r}\right)}{T_{r}^{2}}}t$$

where *CSF* is the severity factor based on the temperature and time contribution, *E_a_* is the reaction activation energy, *R* is the gas constant (8.314 J/(mol K)), *T* is the holding temperature of the process, *T_r_* is the reference temperature and *t* is the holding time. We took 59.2 kJ/mol and 131 °C as activation energy and reference temperature, respectively, related to the T_peak_ of protein denaturation based on the FH equation. In general, a sigmoidal dependency of the pasting parameters was observed as a function of *CSF* (Figure 5A). Similar results were also obtained for WBC (data not shown). However, a few samples did not show the same trend as the others: 80C-24h, 80C-48h and 100C-2h. These samples showed higher PV (i.e., 80C-24h and 80C-48h), higher FV (i.e., 100C-2h) and higher BD (i.e., 80C-24h and 80C-48h) compared to observed trends. For this reason, they were selected for testing in the bread application. Sample 100C-4h was chosen as it displayed a lower PV and BD but similar FV compared to the reference (Figure 5B) while sample 120C-2h showed a general reduction in all pasting parameters. These two samples also followed the trend observed as a function of *CSF*.

### 3.7. Properties of Bread with Selected Dry-Heated CPF

**Figure 6:**
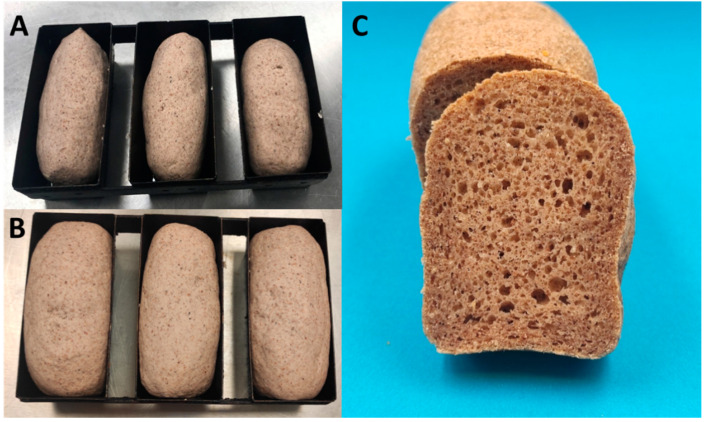
CRC bread dough after mixing and molding in tins (**A**), after proofing (**B**) and the resulting baked bread (**C**).

A bread formulation mainly based on climate-resilient crops (i.e., sorghum flour, cassava flour and CPF) was developed during pre-trials to test the effect of dry heating on bread quality. The bread dough was intended to be processable in conditions similar to wheat dough, as this is critical for implementation in small bakeries in SSA [1]. Therefore, the reference dough was moldable and could be easily shaped by hand to fill the baking tins (Figure 6A,B and Supplementary Video S1). The fermented and baked dough resulted in breads of a pleasant appearance, resembling that of whole meal bread (Figure 6C).

The replacement of native CPF with dry-heated ones did not show any significant effect on SV (Table 3) or on crumb density (data not shown). However, significant effects were observed for the crumb texture based on TPA. All breads with dry-heated CPF showed a significant reduction in crumb hardness compared to the reference, with 100C-2h showing the highest softness, similar to 100C-4h and 120C-2h. Sample 100C-2h also showed the highest cohesiveness and resilience among all breads (*p* < 0.05). A correlation analysis between bread properties and the physicochemical properties of CPF indicated that crumb hardness was positively correlated with the peak temperature of protein denaturation (R^2^ = 0.741; *p* < 0.05). Cohesiveness, springiness and resilience were inversely related to BD (R^2^ = 0.735, R^2^ = 0.799 and R^2^ = 0.695; *p* < 0.05). The observed improvements in crumb texture were quite remarkable, considering that CPF accounted for only 8% of the dry flour ingredients (i.e., sorghum, cassava, CPF and psyllium). These results suggest a significant contribution of CPF in terms of functionality within the bread formulation of this study.

Previous studies on gluten-free breads have shown that protein aggregation during baking has negative effects on bread quality [40], including crumb hardness [44,45]. The addition of proteases in GF breads limited the formation of large protein aggregates, which disrupted the continuity of the starch gel phase [46]. As a result, protease-treated breads from GF flours were characterized by a more cellular microstructure, which confers a softer texture to the crumb. Dry heating induced partial protein denaturation and aggregation prior to baking, which probably made them less reactive during the baking process. From such a perspective, their addition as inert aggregates may have also ensured the continuity of the starch phase.

In recent studies [22,47], it has been suggested that crumb cohesiveness and resilience depend more on starch gelatinization and swelling than on gluten aggregation. In GF breads, the rapid re-association of leached amylose molecules as the starch matrix cools down after baking has been suggested to affect crumb texture [48]. A higher SB associated with the formation of amylose-amylose reassociations (i.e., physical junctions) was related to an increase in crumb hardness and reduced cohesiveness in gluten-free breads based on different rice flour varieties [49]. This mechanism cannot explain the observed changes in the crumb texture in this study. Compared to all other CPF samples, 100C-2h showed an increase in SB, while the resulting breads had the lowest hardness and the highest cohesiveness. Furthermore, no correlations were found between SB and crumb cohesiveness. On the contrary, increasing the amount of starch granules that maintain their integrity during heating, i.e., decreased BD, has been shown to increase the cohesiveness of the crumb by providing mechanical support to the flowing starch paste [50]. This finding agrees with the inverse relation between cohesiveness and BD in this study. In addition to granule rigidity, the continuity and strength of the starch phase upon cooling may have been positively affected by an increased SB for sample 100C-2h, thus explaining the highest value of cohesiveness and resilience observed among all breads.

## 4. Conclusions

In this study, dry heating was investigated as a simple technology to improve CPF functionality for bread-type applications. Against the state of the art, the novelty in our approach was the selection of dry-heating conditions based on a thermodynamic description of sorption behavior of CPF and of biopolymer melting (i.e., starch gelatinization and protein denaturation) in CPF as a function of moisture content. First, protein and starch melting were well described by the FH theory, while sorption properties of CPF and CPPC could be described with the FHFV theory. Next, the effect of dry heating on CPF functionality (i.e., pasting properties and WBC) could be related to temperature-time combinations as described by the CSF using biopolymer phase transitions (i.e., activation energy and melting temperature) as reference conditions. Interestingly, the treatments did not show significant effects on CPF melting endotherms, as the temperature conditions were all well below the peak temperature for starch and protein melting. Specific temperature-time combinations enabled the selective modulation of pasting parameters by increasing PV (i.e., 80 °C for 24–48 h) or FV and SB (i.e., 100 °C for 2 h) compared to the native flour. Finally, the inclusion of dry-heated CPF in a CRC-based bread dough formulation improved crumb softness, cohesiveness and resilience, which are important quality attributes of bread crumb texture. Overall, the results of this study indicate that dry heating of CPF under controlled conditions is an interesting technology for promoting the use of CPF in bread-type products as a technological and nutritionally functional ingredient. Future studies could investigate in more depth the changes in protein and starch components during dry heating and the implications for sensory and nutritional properties of bread-type products (i.e., tin breads and fat breads) enriched with dry-heated CPF.

## Figures and Tables

**Table 1:** Solubility and WBC of native and dry-heated CPF. For the dry-heated CPF samples, the temperature (C) and time (h) treatment conditions are indicated by the codes.

Samples	Solubility (%)	WBC (g/g)
Native	29.1 ^e^ ± 0.4	4.1 ^e^ ± 0.1
80C-2h	27.0 ^d^ ± 0.0	4.1 ^e^ ± 0.1
80C-4h	26.2 ^d^ ± 0.4	3.8 ^de^ ± 0.2
80C-24h	21.9 ^b^ ± 0.1	3.8 ^de^ ± 0.0
80C-48h	21.2 ^b^ ± 0.3	3.6 ^d^ ± 0.0
100C-2h	21.6 ^b^ ± 0.3	3.1 ^bc^ ± 0.1
100C-4h	24.6 ^c^ ± 0.3	2.8 ^ab^ ± 0.3
100C-24h	22.0 ^b^ ± 0.1	3.6 ^cd^ ± 0.2
100C-48h	16.8 ^a^ ± 0.0	2.6 ^a^ ± 0.1
120C-2h	16.4 ^a^ ± 0.6	3.0 ^ab^ ± 0.1
120C-4h	16.6 ^a^ ± 0.0	2.9 ^ab^ ± 0.1
120C-24h	16.4 ^a^ ± 0.1	2.7 ^a^ ± 0.0
120C-48h	17.0 ^a^ ± 1.0	2.6 ^a^ ± 0.0

Different letters in the same column indicate statistically significant differences (*p* < 0.05).

**Table 2:** RVA pasting parameters of native and dry-heated CPF. For the dry-heated CPF samples, the temperature (C) and time (h) treatment conditions are indicated by the codes.

Samples	PV (cP)	HV (cP)	FV (cP)	SB (cP)	T_paste_ (°C)	Peak T. (°C)	BD (cP)
Native	1881.0 ^f^ ± 76.2	1272.3 ^f^ ± 42.5	1953.0 ^f^ ± 67.1	680.7 ^gh^ ± 24.8	77.7 ^b^ ± 0.2	91.8 ± 0.5 ^a^	608.7 ± 44.5 ^c^
80C-2h	1809.0 ^f^ ± 97.1	1300.0 ^fg^ ± 44.8	1983.7 ^f^ ± 49.0	683.7 ^h^ ± 5.5	77.5 ^b^ ± 0.0	93.7 ± 0.6 ^b^	509.0 ± 52.4 ^c^
80C-4h	1888.0 ^f^ ± 84.3	1335.0 ^fgh^ ± 12.3	1990.3 ^f^ ± 5.5	655.3 ^fg^ ± 6.8	77.4 ^b^ ± 0.0	94.7 ± 0.2 ^c^	553.0 ± 96.5 ^c^
80C-24h	2180.7 ^g^ ± 67.7	1351.3 ^gh^ ± 3.8	1974.7 ^f^ ± 5.8	623.3 ^e^ ± 2.1	76.5 ^a^ ± 0.2	94.6 ± 0.1 ^c^	829.3 ± 68.0 ^d^
80C-48h	2289.3 ^g^ ± 26.6	1399.0 ^hi^ ± 16.1	2040.0 ^f^ ± 8.7	641.0 ^ef^ ± 10.4	76.7 ^a^ ± 0.1	94.72 ± 0.1 ^c^	890.3 ± 14.6 ^d^
100C-2h	1647.0 ^e^ ± 84.5	1450.0 ^i^ ± 52.1	2337.7 ^g^ ± 61.8	887.7 ^i^ ± 10.1	77.5 ^b^ ± 0.0	92.4 ± 0.3 ^a^	197.0 ± 33.4 ^b^
100C-4h	1369.0 ^d^ ± 6.1	1144.0 ^e^ ± 14.7	1764.3 ^e^ ± 21.1	620.3 ^e^ ± 6.5	78.6 ^c^ ± 0.0	91.8 ± 0.5 ^a^	225.0 ± 9.0 ^b^
100C-24h	139.0 ^ab^ ± 10.0	131.0 ^b^ ± 10.0	229.0 ^b^ ± 15.5	98.0 ^b^ ± 5.6	-	95.1 ± 0.0 ^c^	8.0 ± 0.0 ^a^
100C-48h	67.0 ^ab^ ± 1.7	62.3 ^ab^ ± 1.2	91.7 ^a^ ± 0.6	29.3 ^a^ ± 0.6	-	95.0 ± 0.0 ^c^	4.7 ± 0.6 ^a^
120C-2h	504.7 ^c^ ± 19.1	493.3 ^d^ ± 16.3	806.0 ^d^ ± 26.6	312.7 ^d^ ± 10.5	84.0 ^d^ ± 0.8	95.0 ± 0.0 ^c^	15.3 ± 6.5 ^a^
120C-4h	218.0 ^b^ ± 7.0	204.0 ^c^ ± 4.6	370.3 ^c^ ± 10.7	166.3 ^c^ ± 6.7	-	95.0 ± 0.0 ^c^	16.7 ± 6.0 ^a^
120C-24h	73.3 ^ab^ ± 5.0	70.0 ^ab^ ± 5.6	101.0 ^a^ ± 7.5	31.0 ^a^ ± 2.0	-	95.0 ± 0.0 ^c^	4.3 ± 2.1 ^a^
120C-48h	36.7 ^a^ ± 2.5	34.0 ^a^ ± 2.6	50.0 ^a^ ± 3.0	16.0 ^a^ ± 1.0	-	95.0 ± 0.0 ^c^	3.0 ± 1.0 ^a^

Different letters in the same column indicate statistically significant differences (*p* < 0.05).

**Table 3:** Bread properties as affected by native and dry-heated CPF. For the dry-heated CPF samples, the temperature (C) and time (h) treatment conditions are indicated by the codes.

	Native	80C-24h	80C-48	100C-2h	100C-4h	120C-2h
SV (mL/g)	1.66 ^b^ ± 0.06	1.51 ^a^ ± 0.07	1.62 ^ab^ ± 0.04	1.56 ^ab^ ± 0.10	1.57 ^ab^ ± 0.08	1.56 ^ab^ ± 0.03
Crumb properties						
Moisture content (%)	49.2 ^a^ ± 0.6	49.7 ^a^ ± 0.4	49.1 ^a^ ± 0.7	49.9 ^a^ ± 0.6	49.2 ^a^ ± 0.7	49.6 ^a^ ± 0.6
Hardness (N)	17.1 ^d^ ± 1.8	15.8 ^cd^ ± 1.7	14.3 ^bc^ ± 1.3	11.6 ^a^ ± 1.3	12.4 ^ab^ ± 1.4	12.1 ^a^ ± 2.1
Cohesiveness	0.63 ^ab^ ± 0.02	0.63 ^ab^ ± 0.02	0.60 ^a^ ± 0.05	0.68 ^c^ ± 0.05	0.66 ^bc^ ± 0.02	0.65 ^bc^ ± 0.02
Springiness	0.90 ^ab^ ± 0.01	0.90 ^ab^ ± 0.01	0.88 ^a^ ± 0.03	0.91 ^bc^ ± 0.01	0.91 ^bc^ ± 0.01	0.92 ^c^ ± 0.03
Resilience	0.31 ^ab^ ± 0.01	0.31 ^ab^ ± 0.02	0.29 ^a^ ± 0.04	0.35 ^c^ ± 0.03	0.33 ^bc^± 0.01	0.31 ^bc^ ± 0.01

Different letters in the same row indicate statistically significant differences (*p* < 0.05).

## Data Availability

The data presented in this study are available in article.

## Supplementary Materials

The following supporting information can be downloaded at: https://www.mdpi.com/article/10.3390/foods11111554/s1, Figure S1: Sorption isotherm of CPF and CPPC; Figure S2: DSC thermograms for dry heating of CPF at 80 °C (A), 100 °C (B) and 120 °C (C). Native CPF is shown in each graph by the red line. Dry-heating time (green lines) from 2 h to 48 h (from bottom to top); Table S1: Melting temperatures and enthalpies of transitions for native and dry-heated CPF. T_peak,1_ is associated with starch gelatinization and T_peak,2_ to protein denaturation; Video S1: Molding of dough.
