# Supplementary material for: Dry Heating of Cowpea Flour below Biopolymer Melting Temperatures Improves the Physical Properties of Bread Made from Climate-Resilient Crops
Source: Foods. 2022 May 25;11(11):1554. doi: 10.3390/foods11111554 (PMC9180669; doi:10.3390/foods11111554)
Supplement: Supplementary file 1 [file foods-11-01554-s001.zip › foods-1743119-supplementary/Supplementary material.pdf]

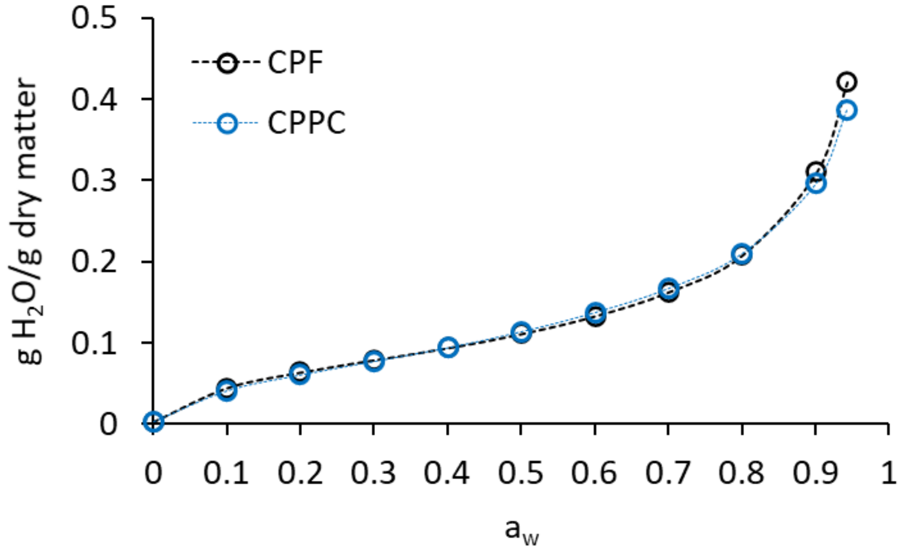

Figure S1. Sorption isotherm of CPF and CPPC.

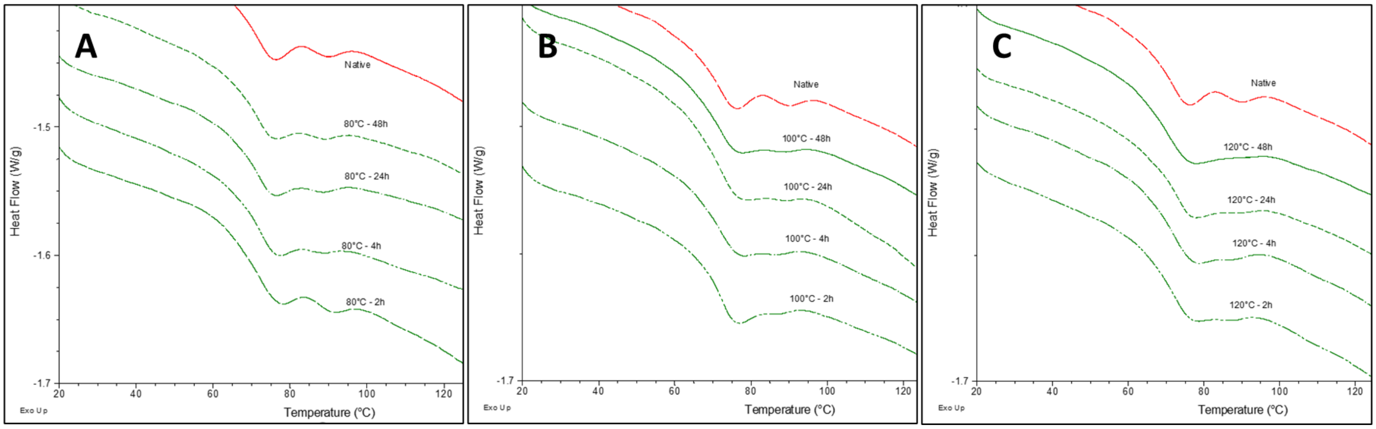

Figure S2. DSC thermograms for dry-heating of CPF at 80 °C (A), 100 °C (B) and 120 °C (C). Native CPF is shown in each graph by the red line. Dry-heating time (green lines) from 2 h to 48 h (from bottom to top).

Table S1. Melting temperatures and enthalpies of transitions for native and dry-heated CPF. $T_{peak,1}$ is associated to starch gelatinization and $T_{peak,2}$ to protein denaturation.

Samples	Enthalpy (J/g)	T_{onset} (°C)	$T_{peak,1}$ (°C)	$T_{peak,2}$ (°C)
**Native**	$8.7^{ab} \pm 0.2$	$64.6^{ab} \pm 0.9$	$75.7^{ab} \pm 0.2$	$89.5^{c} \pm 0.6$
**80C-2h**	$10.1^{abc} \pm 0.8$	$64.1^{ab} \pm 1.2$	$76.7^{cd} \pm 0.4$	$89.4^{c} \pm 1.3$
**80C-4h**	$9.0^{ab} \pm 0.2$	$65.7^{b} \pm 0.4$	$76.3^{abcd} \pm 0.2$	$88.5^{abc} \pm 1.0$
**80C-24h**	$10.5^{bc} \pm 1.2$	$64.7^{ab} \pm 1.3$	$75.5^{a} \pm 0.1$	$89.1^{bc} \pm 1.6$
**80C-48h**	$8.9^{ab} \pm 0.5$	$65.0^{ab} \pm 0.3$	$75.5^{a} \pm 0.1$	$87.6^{abc} \pm 1.1$
**100C-2h**	$9.5^{abc} \pm 0.7$	$64.1^{ab} \pm 1.2$	$76.0^{abc} \pm 0.1$	$87.7^{abc} \pm 0.9$
**100C-4h**	$8.8^{ab} \pm 0.4$	$65.7^{b} \pm 0.4$	$76.5^{bcd} \pm 0.2$	$86.8^{abc} \pm 0.2$
**100C-24h**	$9.8^{abc} \pm 0.7$	$64.7^{ab} \pm 1.3$	$76.6^{cd} \pm 0.2$	$87.7^{abc} \pm 0.7$
**100C-48h**	$11.0^{c} \pm 1.0$	$65.0^{ab} \pm 0.3$	$76.6^{bcd} \pm 0.4$	-
**120C-2h**	$8.4^{a} \pm 0.2$	$65.3^{ab} \pm 0.3$	$76.6^{cd} \pm 0.6$	$86.1^{a} \pm 0.3$
**120C-4h**	$9.1^{abc} \pm 0.3$	$64.7^{ab} \pm 0.5$	$77.2^{d} \pm 0.2$	$86.6^{ab} \pm 0.8$
**120C-24h**	$9.7^{abc} \pm 1.1$	$63.9^{ab} \pm 1.7$	$76.3^{abcd} \pm 0.5$	-
**120C-48h**	$11.0^{c} \pm 0.5$	$62.5^{a} \pm 1.0$	$76.3^{abcd} \pm 0.5$	-
